# CogStrat: web-based tools for predicting incident cognitive impairment in cognitively normal older adults

**DOI:** 10.1007/s00702-026-03163-7

**Published:** 2026-05-26

**Authors:** Adolfo Jiménez-Huete, Jimmy Santiago Ulloa Bravo, Antonio Martín-Bastida, Teresa Rognoni, Genoveva Montoya-Murillo, María Cruz Rodríguez-Oroz, Mario Riverol

**Affiliations:** 1 Department of Neurology, Clínica Universidad de Navarra, C/ Marquesado de Santa Marta, 1, 28027 Madrid and Pamplona, Spain; 2 Network Center for Biomedical Research in Neurodegenerative Diseases (CIBERNED), Madrid, Spain; 3 Instituto de Investigación Sanitaria de Navarra (IdisNA), Pamplona, Spain

**Keywords:** Cognitive impairment, Predictive models, Cox proportional hazards regression, Montreal cognitive assessment, Biomarkers, User–computer interface

## Abstract

Early identification of individuals at increased risk of cognitive impairment is crucial for timely intervention. We developed and evaluated Cox proportional hazards models and translated them into web-based applications to predict incident cognitive impairment in cognitively normal older adults using clinical data, with optional biomarker inputs. Data were drawn from cognitively unimpaired participants in the Alzheimer’s Disease Neuroimaging Initiative (ADNI). Models included demographic and neuropsychological variables, with optional genetic, neuroimaging, and fluid (plasma and CSF) biomarkers. Performance was assessed using the C-index, calibration slope, and decision curve analysis, with internal validation via bootstrap resampling. Key models were externally validated in the Harvard Aging Brain Study. The ADNI sample comprised 729 participants (median age 73 years; 45% male). A clinical model including age, education, Montreal Cognitive Assessment, and delayed word recall showed acceptable discrimination (C-index 0.721; CI 0.672–0.772) and strong calibration (slope 0.940). Adding biomarkers improved performance, with the highest discrimination observed for a model including FDG and florbetapir PET uptake (C-index 0.805; CI 0.759–0.886). Discrimination remained stable after internal and external validation, while calibration was weaker for most plasma biomarkers. Five web applications (CogStrat) were developed using clinical data alone or combined with hippocampal volume, CSF tau/Aβ42, plasma neurofilament light chain, or FDG and florbetapir PET. These tools provide individualized risk group classifications, 5-year risk estimates, and 10-year cumulative incidence curves. CogStrat translates prognostic models into freely accessible, interactive tools that support risk stratification of cognitive impairment in cognitively normal older adults.

**Supplementary Information:**

The online version contains supplementary material available at https://doi.org/10.1007/s00702-026-03163-7.

## Introduction

Dementia is one of the most pressing public health challenges worldwide and a major priority for the World Health Organization (2022). More than 50 million people were living with dementia in 2020, and its prevalence is projected to nearly double every 20 years. Mild cognitive impairment (MCI) is even more common, affecting 6–25% of individuals aged 65 years and older (Dunne et al. 2021). Alzheimer’s disease (AD) accounts for roughly two-thirds of dementia cases in developed countries (Alzheimer’s Association 2024).

Historically, the diagnostic approach to dementing disorders has been largely passive, relying on patients or relatives to seek medical attention once symptoms become evident (WHO 2019). The recent development and regulatory approval of disease-modifying therapies targeting amyloid pathology has challenged this paradigm. Clinical trials of anti-amyloid monoclonal antibodies, such as lecanemab and donanemab, have demonstrated their ability to reduce amyloid burden and to slow cognitive and functional decline in early-stage AD, establishing proof of principle for disease modification (Fox et al. 2025). These advances mark a shift toward biologically targeted treatment and have led to increasing consensus regarding their clinical efficacy in appropriately selected patients. As these therapies are most effective in the earliest stages of disease, there is a growing need for methods that support early identification of individuals at increased risk of cognitive decline and facilitate timely clinical decision-making.

A wide range of factors have been investigated for the early detection or prediction of AD and cognitive impairment. These include demographic characteristics, vascular and lifestyle risk factors, neuropsychological test performance, genetic markers such as *APOE* genotype, fluid biomarkers measured in blood or cerebrospinal fluid (CSF), and structural or functional neuroimaging measures (Jungwirth et al. 2009; Alexopoulos et al. 2018; Marshe et al. 2019; Wilson et al. 2019; Frisoni et al. 2025). While CSF biomarkers and advanced neuroimaging techniques provide valuable information, their use is often limited by cost, accessibility, and invasiveness (Frisoni et al. 2025). Blood-based biomarkers represent a promising alternative; however, their routine implementation may also be constrained by issues related to availability, standardization, and interpretation, particularly outside specialized centers (Hansson et al. 2023).

Several studies have developed multivariable models that integrate these sources of information to estimate the risk of cognitive impairment among cognitively unimpaired individuals (Stephan et al. 2010; Tang et al. 2015; Mohanannair Geethadevi et al. 2023; Brain et al. 2024). The ABIDE project, for instance, developed models incorporating cognitive performance, CSF biomarkers, and structural MRI to predict progression to MCI or dementia in memory clinic cohorts (van Maurik et al. 2019). Although these models provide useful prognostic information, their discrimination is typically only moderate, reflecting the complex and heterogeneous nature of cognitive disorders (Jellinger 2022). In this context, stratifying individuals into clinically meaningful risk groups may be more robust than attempting highly precise absolute risk prediction. Cox proportional hazards (PH) models are well suited to this purpose because they estimate relative event rates over time, enabling clinically meaningful risk stratification across follow-up periods and allowing flexible incorporation of predictors (Sutradhar and Austin 2018).

Despite these advances, important gaps remain that limit the clinical applicability of existing prediction models. Many studies have focused primarily on discrimination, with less systematic evaluation of calibration, which is essential for accurate risk estimation in individual patients. External validation has been performed inconsistently, raising concerns about generalizability across populations and settings. In addition, most models have not been implemented as accessible tools that allow clinicians to obtain individualized risk estimates at the point of care. Finally, existing approaches often rely on fixed sets of predictors and do not provide flexible frameworks that can accommodate different levels of data availability, from routine clinical variables to advanced biomarker assessments. Together, these limitations hinder the translation of prognostic models into routine clinical practice.

Building on prior work identifying cognitive performance profiles associated with increased risk of cognitive decline in cognitively normal older adults (Jiménez-Huete et al. 2024), we used data from the Alzheimer’s Disease Neuroimaging Initiative (ADNI; https://adni.loni.usc.edu) to develop and internally validate a set of Cox PH models for predicting incident cognitive impairment. The models incorporate demographic and neuropsychological variables, with optional inclusion of structural neuroimaging and fluid (plasma and CSF) biomarkers. Key models were externally validated in the Harvard Aging Brain Study (HABS; https://habs.mgh.harvard.edu).

To facilitate translation into clinical and research practice, five models were implemented as web-based applications (CogStrat), enabling individualized risk estimation and risk stratification at the point of care. By providing a tiered set of models with progressively richer inputs, these tools can be adapted to different settings and levels of diagnostic resources.

## Methods

### Study population and data availability

The main study sample was drawn from ADNI (https://adni.loni.usc.edu, accessed June 15, 2024). We included participants from the ADNI-1, ADNI-GO, ADNI-2, and ADNI-3 cohorts who were cognitively unimpaired at baseline and had at least one follow-up visit. In addition, data on novel plasma biomarkers were obtained from ADNI-4. The ADNI was launched in 2003 as a public–private partnership, led by Michael W. Weiner. Its primary goal has been to test whether MRI, positron emission tomography (PET), other biological markers, and clinical and neuropsychological assessment can be combined to measure the progression of MCI and early AD (www.adni-info.org).

The HABS was used for external validation of the models fitted with the ADNI datasets (HABS data release 2.0; https://habs.mgh.harvard.edu; accessed August 20, 2024) (Dagley et al. 2017).

The ADNI and HABS datasets can be accessed upon reasonable request to the ADNI and HABS committees.

### Ethical considerations

The study was conducted in accordance with the Declaration of Helsinki and approved by the Research Ethics Committee of the Universidad de Navarra (Project ID: 2025.112).

### Baseline variables and time of follow-up

The variables analyzed at baseline included:

- Demographic data: age, sex, and years of education.
- Cardiovascular risk.
- Neuropsychological variables: Mini-Mental State Examination (MMSE) (Folstein et al. 1975), Montreal Cognitive Assessment (MoCA) (Nasreddine et al. 2005), delayed word recall from the ADAS-Cog (Rosen et al. 2006), categorical fluency (Ruff et al. 1996), Trail Making Test parts A and B (TMT-A and TMT-B) (Reitan 1958), and the Geriatric Depression Scale (GDS) (Yesavage et al. 1982).
- Genetic variables: *APOE* ε4 carrier status (APOE4) and the Desikan polygenic hazard score (PHS) (Desikan et al. 2017).
- Plasma biomarkers: phosphorylated tau at threonine 217 (p-tau217), amyloid-beta 1-42 (Aβ42)/amyloid-beta 1-40 (Aβ40), p-tau217/Aβ42 and neurofilament light chain (NfL) (Hansson et al. 2023).
- CSF biomarkers: Aβ42, phosphorylated tau at threonine 181 (p-tau181), total tau, p-tau181/Aβ42 and total tau/Aβ42 (Hazan et al. 2022).
- Neuroimaging variables: bilateral hippocampal volume estimated with MRI (Rao et al. 2023), meta-ROI [18F]fluorodeoxyglucose (FDG) uptake on PET and florbetapir average cortical uptake on PET (Landau et al. 2011, 2013).

All predictors were coded as continuous variables, except for sex, cardiovascular risk and *APOE* ε4 carrier status, which were evaluated as binary variables.

The outcome of interest was incident cognitive impairment, defined as progression from normal cognition to either MCI or dementia. Diagnoses in ADNI are established using standardized, protocol-driven procedures, as specified in the ADNI manuals (https://adni.loni.usc.edu). At each study visit, participants undergo a comprehensive clinical and neuropsychological assessment that includes global cognitive measures (e.g., MMSE), domain-specific neuropsychological testing (covering memory, language, and executive function), and evaluation of functional status using instruments such as the CDR and the FAQ. Diagnostic classification is determined at each visit through a multidisciplinary consensus process involving neurologists, neuropsychologists, and other trained clinicians, integrating all available information.

In ADNI, MCI is defined by the presence of a subjective memory complaint reported by the participant or an informant, together with objective cognitive impairment (typically in the memory domain) documented by performance below education-adjusted norms on standardized tests, while functional abilities are largely preserved or only minimally impaired (corresponding to a CDR global score of 0.5 and minimal impairment on the FAQ). Dementia is defined by cognitive impairment affecting at least two domains, accompanied by clinically significant functional decline that interferes with independence in daily life (corresponding to a CDR global score ≥ 1). Diagnosis also requires evidence that cognitive deficits represent a decline from a previous level of functioning and are not better explained by delirium, major psychiatric disorders, or other non-neurodegenerative conditions.

We defined time of follow-up as the time from study inclusion to either conversion to cognitive impairment (MCI or dementia) or the last recorded visit.

### Preprocessing and univariate evaluation of predictor variables

Predictor distributions were examined using summary statistics and density plots. Continuous variables were transformed to approximate symmetry, using logarithmic or exp(− 0.5) transformations for right-skewed variables and squared transformations for left-skewed variables. Continuous predictors were also standardized to have a mean of 0 and a standard deviation of 1, allowing direct comparison of effect sizes. In addition, variables were coded so that higher values consistently reflected greater risk of cognitive impairment. This required reversing the direction of variables in which higher values indicate better cognitive function, such as MMSE and MoCA.

### Development of multivariable Cox models for predicting cognitive impairment

Multivariable Cox PH models were developed and evaluated following a strategy consistent with the TRIPOD+AI statement (Collins et al. 2024): (1) Age and years of education were included in all models based on subject-matter knowledge. (2) A clinical model was selected using elastic net regression applied to demographic and neuropsychological variables (Witten and Tibshirani 2009). (3) The incremental value of biomarkers was assessed by comparing models that included each biomarker with the clinical model fitted to the same dataset. Model comparisons were conducted using likelihood ratio tests (LRT) and the net reclassification index (NRI) (Pencina et al. 2008). (4) Final model selection considered discrimination, calibration, sample size and number of events, accessibility, and internal and external validation (Harrell et al. 1996).

### Discrimination and calibration of predictive models

Model discrimination was evaluated using the concordance index (C-index), which quantifies the ability of a model to correctly rank individuals according to their risk of cognitive impairment.

Model calibration was assessed using the slope of the calibration curve, which reflects the agreement between predicted and observed risks.

### Internal and external validation of predictive models

Internal validation was performed using bootstrap resampling (B = 1000) to correct for potential overfitting. Model performance was summarized using the optimism-corrected C-index (Harrell et al. 1996).

External validation was conducted by calculating the C-index in the HABS cohort. Since the HABS lacks MoCA scores, we estimated them from MMSE scores (Fasnacht et al. 2023). Based on the availability of predictors, we were able to externally validate the models that included clinical data, APOE4, hippocampal volume, and FDG-PET, but not those incorporating fluid biomarkers or florbetapir-PET.

### Kaplan–Meier cumulative incidence curves for cognitive impairment stratified by risk group

We constructed Kaplan–Meier curves to visualize the cumulative incidence of cognitive impairment across the risk groups defined by the Cox models. Risk groups were defined by calculating individual linear predictor values and stratifying participants into tertiles corresponding to low, medium, and high risk groups.

### Decision curve analysis

To further evaluate model performance from a clinical perspective, we performed decision curve analysis (DCA) at 5 years (Sjoberg 2025). DCA curves plot net benefit on the y-axis against threshold probability on the x-axis. The net benefit of each model is compared to two default clinical strategies: the “treat-all” and “treat-none” strategies. In this context, these strategies correspond to considering all individuals cognitively impaired or considering no individuals impaired, regardless of the model’s predictions.

### CogStrat applications

Selected models were implemented as web applications (CogStrat) using the shiny package (Chang et al. 2024). Users input predictor values and receive a risk category, a 5-year risk estimate with 95% confidence interval (CI), and 10-year cumulative incidence curves for the current case and an average case (with median values of predictors). A warning is displayed for education-adjusted MoCA scores below 24, suggesting current cognitive impairment (Carson et al. 2018).

### Statistical software

Statistical analyses were done with R (version 4.2.2) (R Core Team 2021) and the following packages: ADNIMERGE (0.0.1) (Alzheimer’s Disease Neuroimaging Initiative 2023), rms (6.8-0) (Harrell 2024), survival (3.5-0) (Therneau 2023), survminer (0.4.9) (Kassambara 2021), dcurves (0.5.19) (Sjoberg 2025) and shiny (1.10.0) (Chang et al. 2024).

## Results

### Study population

We identified 729 participants from the ADNI cohorts with normal cognitive function at baseline, including 244 (33%) with cognitive complaints and 485 (67%) without cognitive complaints. Participants had a median age of 73 years, 45% were male, and the median years of education was 16. Baseline cognitive performance was high overall, with a median MMSE score of 29. Among these individuals, 146 (20%) developed cognitive impairment over a median follow-up time of 1469 days. The majority of events corresponded to progression to MCI (n = 140), while only a small number of participants progressed directly to dementia (n = 6). The summary statistics are shown in Table 1.

**Table 1:** Characteristics of the Alzheimer’s Disease Neuroimaging Initiative (ADNI) sample of cognitively unimpaired participants (n = 729), stratified by conversion to cognitive impairment (mild cognitive impairment or dementia)

Variables	Conversion = No	Conversion = Yes	All participants
	n = 583	n = 146	n = 729
Time of follow-up (days)	1465 (788, 2346)	1483 (738, 2516)	1469 (777, 2386)
Age (years)	72 (68, 77)	74 (71, 78)	73 (69, 77)
Sex (male)	252 (43%)	78 (53%)	330 (45%)
Education (years)	16 (15, 18)	16 (15, 18)	16 (15, 18)
Memory complaints	208 (36%)	36 (25%)	244 (33%)
Cardiovascular risk factors	246 (65%)	94 (63%)	340 (67%)
MMSE (0–30)	29 (29, 30)	29 (28, 30)	29 (29, 30)
MoCA (0–30)	26 (24, 28)	23 (22, 25)	26 (24, 28)
Delayed word recall (0–10)	8 (7, 9)	7 (5, 8)	8 (6, 9)
Categorical fluency (animals)	21 (18, 25)	20 (16, 23)	21 (17, 24)
TMT-A (s)	30 (25, 38)	34 (29, 42)	31 (26, 38)
TMT-B (s)	70 (55, 91)	83 (64, 105)	73 (56, 93)
Geriatric Depression Scale	0 (0, 1)	1 (0, 2)	0 (0, 1)
*APOE* ε4 carrier	166 (29%)	53 (37%)	219 (31%)
Polygenic hazard score	− 0.15 (− 0.41, 0.37)	− 0.09 (− 0.32, 0.79)	− 0.13 (− 0.39, 0.48)
p-tau217 in plasma (pg/mL)	0.11 (0.07, 0.15)	0.17 (0.11, 0.33)	0.12 (0.08, 0.21)
Aβ42/Aβ40 in plasma	0.09 (0.08, 0.10)	0.09 (0.08, 0.11)	0.09 (0.08, 0.10)
p-tau217/Aβ42 in plasma	0.00 (0.00, 0.01)	0.01 (0.01, 0.02)	0.01 (0.00, 0.01)
NfL in plasma (pg/mL)	30 (23, 38)	39 (28, 44)	31 (24, 41)
Aβ42 in CSF (pg/mL)	1053 (799, 1406)	872 (623, 1263)	994 (731, 1360)
p-tau181 in CSF (pg/mL)	19 (15, 25)	21 (17, 31)	20 (16, 26)
Total tau in CSF (pg/mL)	212 (173, 268)	234 (186, 335)	215 (176, 280)
p-tau181/Aβ42 in CSF	0.02 (0.01, 0.03)	0.03 (0.01, 0.04)	0.02 (0.01, 0.03)
Tau/Aβ42 in CSF	0.17 (0.13, 0.30)	0.31 (0.16, 0.42)	0.19 (0.13, 0.34)
Hippocampal volume (µL)	7598 (7040, 8147)	7202 (6604, 7691)	7506 (6941, 8011)
FDG-PET (SUVR)	1.29 (1.22, 1.37)	1.24 (1.15, 1.32)	1.28 (1.20, 1.36)
Florbetapir-PET (SUVR)	1.05 (0.99, 1.20)	1.21 (1.03, 1.32)	1.07 (0.99, 1.26)

A substantial proportion of data was missing for fluid biomarkers and PET measures; the datasets used to fit the predictive models are shown in Supplementary Table 1. Descriptive statistics are reported as median (interquartile range) or frequency (percentage). Delayed word recall refers to the number of words recalled on the ADAS-Cog task. Hippocampal volume denotes bilateral hippocampal volume derived from MRI. FDG-PET reflects glucose uptake in AD-related regions (meta-ROI). Florbetapir-PET represents average cortical amyloid uptake

*ADAS-Cog* Alzheimer’s Disease Assessment Scale-Cognitive subscale, *CSF* cerebrospinal fluid, *FDG* [18F]Fluorodeoxyglucose, *MMSE* Mini-Mental State Examination, *MoCA* Montreal Cognitive Assessment, *n* sample size, *NfL* neurofilament light chain, *PET* positron emission tomography, *p-tau181* phosphorylated tau at 181, *p-tau217* phosphorylated tau at 217, *SUVR* standardized uptake value ratio, *TMT-A* Trail Making Test part A, *TMT-B* Trail Making Test part B

Models were fitted on different subsets of the ADNI sample depending on data availability (Supplementary Table 1). Due to substantial missingness in biomarker measures, with training dataset sizes ranging from 613 participants for the clinical model to 181 for the PET model, multiple imputation was not performed. Demographic characteristics were generally similar across subsets, although times of follow-up tended to be longer in datasets that included CSF biomarkers and PET measures.

External validation was performed in the HABS cohort (n = 287), comprising cognitively unimpaired older adults with a median age of 78 years, 41% male, and a median of 16 years of education. During follow-up, 23 participants (8%) developed cognitive impairment. Baseline cognitive performance was high overall (median MMSE 29), and biomarker data, including hippocampal volume and PET measures, were available in most participants (Supplementary Table 2). HABS participants were slightly older than those in the ADNI sample (median age 78 vs. 73 years), but global cognitive function, years of education, and sex distribution were similar.

### Predictor variables requiring preprocessing

Several predictors showed right-skewed distributions, including GDS, TMT-A, TMT-B, CSF tau measures, plasma biomarkers, the PHS, and florbetapir uptake, and were transformed using logarithmic or exp(− 0.5) functions. Years of education, MMSE, MoCA, and delayed word recall were left-skewed and were squared. All transformed variables were standardized and aligned so that higher values indicated higher risk.

### Univariate hazard ratios for predictor variables

**Fig. 1:**
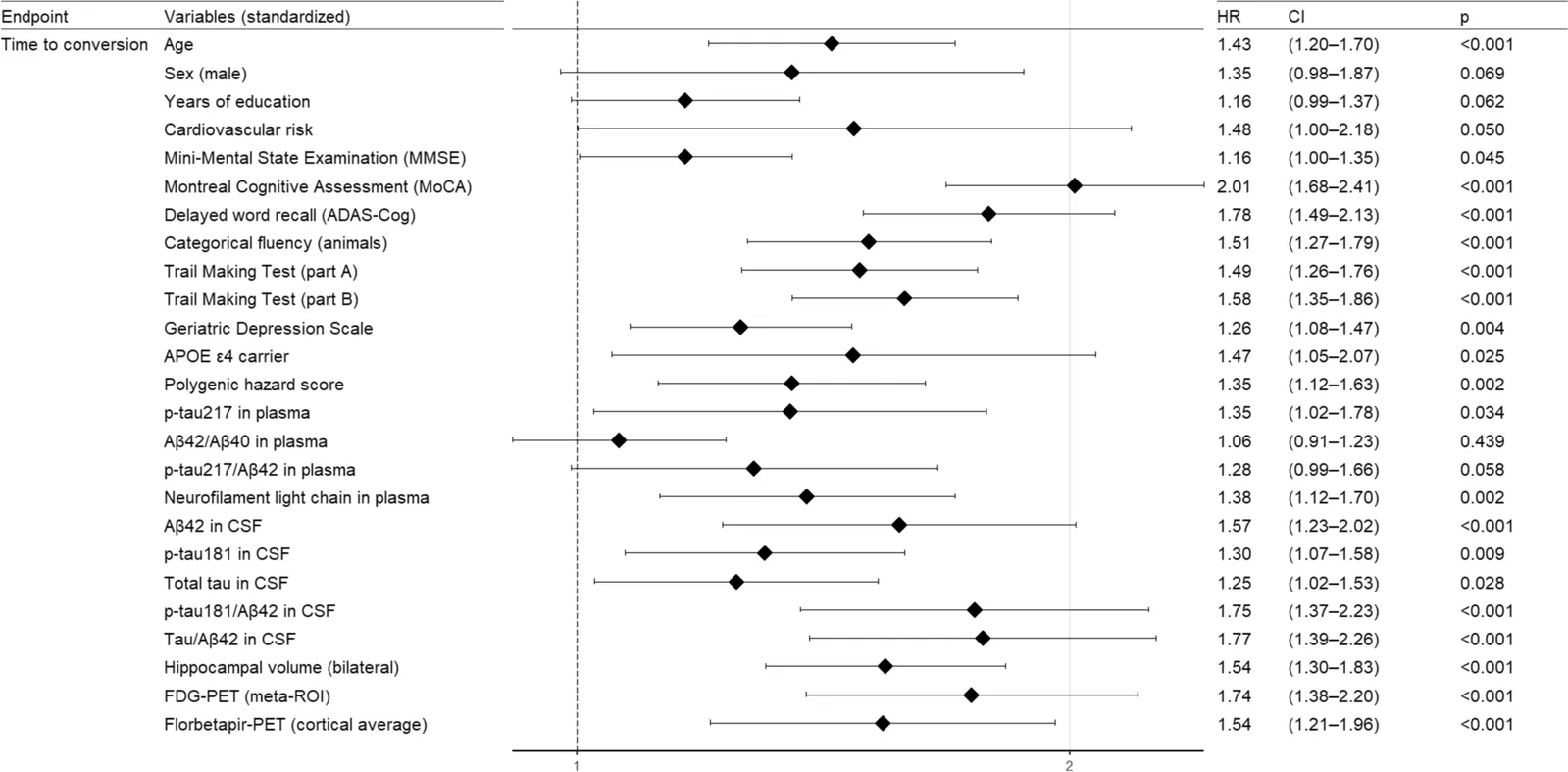
Hazard ratios (HRs) for cognitive impairment of predictor variables, including demographic, neuropsychological, genetic, fluid biomarker (plasma and CSF) and neuroimaging data. Quantitative variables were transformed and standardized to allow their direct comparison, where high values indicate a higher risk of cognitive impairment

Univariate Cox PH models showed strong associations between incident cognitive impairment and older age, lower MoCA scores, poorer delayed word recall, and longer completion times on TMT-B (HR 1.58–2.01, Fig. 1). Genetic risk factors showed moderate effects (HR 1.35–1.47). Among fluid biomarkers, plasma NfL and p-tau217 were the strongest plasma predictors (HR 1.38 and 1.35, respectively), while CSF tau/Aβ42 and p-tau/Aβ42 exhibited the largest overall effects (HR 1.75–1.77). Neuroimaging markers, including hippocampal atrophy, FDG-PET hypometabolism, and increased amyloid burden on florbetapir-PET, were also robustly associated with risk (HR 1.54–1.74).

### Development and internal validation of multivariable Cox models for predicting cognitive impairment

According to our pre-specified strategy, we first obtained a clinical model using elastic net regression that included age, years of education, MoCA, and delayed word recall. Schoenfeld’s residuals test indicated that this model violated the PH assumption due to the delayed word recall variable (*p* = 0.014). This issue was resolved by applying a restricted cubic spline with four knots to this variable. Next, we developed a series of Cox models by incorporating additional predictors. The performance of the models is shown in Table 2, and their components are presented in Table 3.

**Table 2:** Performance of Cox proportional hazards models for predicting incident cognitive impairment in cognitively normal older adults

Model	C-index (95% CI)	Optimism-corrected C-index	Optimism-corrected delta C-index	LRT *p* value	NRI *p* value	Externally validated C-index	Calibration slope
**Clinical model**							
Clinical data (age, education, MoCA, delayed recall)	0.721 (0.672, 0.772)	0.711	–	–	–	0.734	0.940
**Clinical plus genetic models**							
Clinical data plus APOE4	0.725 (0.685, 0.779)	0.713	0.001	0.060	0.292	0.733	0.916
Clinical data plus PHS	0.725 (0.681, 0.775)	0.704	0.019	0.003	0.226	–	0.865
**Clinical plus plasma biomarker models**							
Clinical data plus p-217tau	0.683 (0.605, 0.796)	0.621	0.006	0.307	0.890		0.518
Clinical data plus Aβ42/Aβ40	0.682 (0.608, 0.781)	0.632	0.016	0.028	0.492	–	0.545
Clinical data plus p-217tau/Aβ42	0.674 (0.593, 0.774)	0.612	− 0.003	0.817	0.999	–	0.490
Clinical data plus NfL	0.739 (0.681, 0.817)	0.709	0.012	0.039	0.140	–	0.836
**Clinical plus CSF biomarker models**							
Clinical data plus Aβ42	0.717 (0.636, 0.807)	0.685	0.045	0.005	0.033	–	0.778
Clinical data plus p181tau	0.685 (0.637, 0.771)	0.650	− 0.005	0.313	0.439	–	0.799
Clinical data plus tau	0.686 (0.632, 0.765)	0.657	− 0.006	0.468	0.545	–	0.824
Clinical data plus p-181tau/Aβ42	0.710 (0.654, 0.799)	0.666	0.039	0.003	0.020	–	0.774
Clinical data plus tau/Aβ42	0.715 (0.631, 0.800)	0.682	0.042	0.002	0.007	–	0.784
**Clinical plus neuroimaging models**							
Clinical data plus hippocampal volume	0.751 (0.701, 0.805)	0.735	0.011	0.000	0.492	0.716	0.908
Clinical data plus PET-FDG	0.721 (0.652, 0.785)	0.705	0.004	0.065	0.326	0.637	0.893
Clinical data plus PET-florbetapir	0.765 (0.713, 0.827)	0.748	0.016	0.003	0.140	–	0.840
Clinical data plus FDG and florbetapir	0.805 (0.759, 0.886)	0.768	0.034	0.003	0.047	–	0.791

The C-index in the second column is the apparent C-index, calculated on the training dataset. The optimism-corrected C-index was obtained via bootstrap resampling (B = 1000). The optimism-corrected delta C-index represents the difference between the optimism-corrected C-index of the biomarker-inclusive model and its corresponding clinical model. The externally validated C-index was calculated by applying the fitted model to an independent dataset (Harvard Aging Brain Study)

*Aβ40* amyloid beta 1-40, *Aβ42* amyloid beta 1-42, *APOE4* APOE ε4 carrier, *C-index* concordance index, *95% CI* 95% confidence interval calculated with bootstrap, *CSF* cerebrospinal fluid, *FDG* [18F]Fluorodeoxyglucose, *LRT* likelihood ratio test, *NRI* net reclassification index, *MoCA* Montreal Cognitive Assessment, *PET* positron emission tomography, *p-tau181* phosphorylated tau at threonine 181, *p-tau217* phosphorylated tau at threonine 217

**Table 3:** Cox proportional hazards models for risk stratification of incident cognitive impairment in cognitively normal older adults

Variable	HR (95% CI)	*p*-value
**Clinical model**		
Age (years)	1.14 (0.93, 1.41)	0.214
Education (years)	1.06 (0.89, 1.27)	0.501
MoCA	1.73 (1.42, 2.10)	< 0.001
Delayed word recall (Q1 vs. median)	0.79 (0.64, 0.96)	< 0.001
Delayed word recall (Q3 vs. median)	1.49 (1.23, 1.79)	< 0.001
**Clinical plus genetic models**		
*APOE* ℇ4 carrier	1.44 (0.99, 2.10)	0.056
Polygenic hazard score	1.38 (1.11, 1.70)	0.003
**Clinical plus plasma biomarker models**		
p-tau217_in plasma	1.20 (0.85, 1.69)	0.310
Aβ42/Aβ40 in plasma	1.22 (1.01, 1.48)	0.036
p-tau217/Aβ42 in plasma	1.04 (0.76, 1.42)	0.818
Neurofilament light chain in plasma	1.40 (1.04, 1.88)	0.027
**Clinical plus CSF biomarker models**		
Aβ42 in CSF	1.50 (1.13, 2.00)	0.005
p-tau181 in CSF	1.13 (0.90, 1.41)	0.307
Tau in CSF	1.09 (0.87, 1.37)	0.466
p-tau181/Aβ42 in CSF	1.55 (1.15, 2.09)	0.004
Tau/Aβ42 in CSF	1.58 (1.18, 2.13)	0.002
**Clinical plus neuroimaging models**		
Hippocampal volume	1.44 (1.17, 1.76)	< 0.001
FDG-PET	1.29 (0.98, 1.69)	0.066
Florbetapir-PET	1.47 (1.15, 1.89)	0.002
FDG in combined PET model	1.69 (1.13, 2.54)	0.011
Florbetapir in combined PET model	1.48 (1.08, 2.02)	0.015

Hazard ratios (HRs) for clinical variables are shown for the clinical model; HRs for added biomarkers are shown for the remaining models

All variables were transformed and standardized prior to model fitting such that higher values indicate greater risk of cognitive impairment. Models including biomarkers also adjust for clinical variables, although the corresponding HRs are not shown for brevity (see eTable 1 for full information). HRs for delayed word recall are presented using the median value as the reference, rather than restricted cubic splines, to facilitate interpretation. The combined PET model includes both FDG-PET and florbetapir-PET as predictors

*Aβ40* amyloid beta 1-40, *Aβ42* amyloid beta 1-42, *95% CI* 95% confidence interval, *CSF* cerebrospinal fluid, *FDG* [18F]Fluorodeoxyglucose, *HR* hazard ratio, *MoCA* Montreal Cognitive Assessment, *PET* positron emission tomography, *p-tau181* phosphorylated tau at threonine 181, *p-tau217* phosphorylated tau at threonine 217, *Q1* first quartile, *Q3* third quartile

The clinical model showed acceptable discrimination (apparent C-index 0.721; optimism-corrected C-index 0.711) and excellent calibration (slope 0.940).

The addition of APOE4 did not change discrimination relative to the clinical model. Inclusion of the PHS was associated with a modest increase in discrimination (delta C-index 0.019) and improved model fit, without significant changes in reclassification indices.

Plasma biomarker models including p-tau217, Aβ42/Aβ40, or p-tau217/Aβ42 showed small or no improvement in discrimination over the clinical model and exhibited poor calibration. Plasma NfL was the best-performing plasma biomarker, yielding a modest improvement in discrimination (delta C-index 0.012) and significantly enhancing model fit while maintaining acceptable calibration.

Models including CSF Aβ42, tau/Aβ42 and p-tau181/Aβ42 were associated with larger gains in discrimination (delta C-index 0.039–0.045), significant improvements in model fit and reclassification, and acceptable calibration. Among these models, the model including tau/Aβ42 showed the largest improvement in reclassification and the best calibration.

Neuroimaging biomarkers provided consistent incremental predictive value. The addition of hippocampal volume significantly improved discrimination (delta C-index 0.011) and model fit, while maintaining a calibration slope close to unity (0.908). The model with florbetapir-PET also enhanced discrimination, although without significant improvements in reclassification. The combined FDG and florbetapir PET model achieved the highest overall performance (apparent C-index 0.805; optimism-corrected C-index 0.768), with significant gains in model fit and reclassification, and acceptable calibration.

### External validation of multivariable Cox models for predicting cognitive impairment

External validation in the HABS cohort confirmed the robustness of the clinical model, which showed a C-index of 0.734 (Table 3, last column). The model including APOE4 yielded a similar C-index (0.733). The model incorporating hippocampal volume also performed well externally (C-index 0.716), whereas the model including FDG-PET uptake showed lower discrimination than in the training dataset (apparent C-index 0.721; external C-index 0.637). Models incorporating plasma or CSF biomarkers were not externally validated due to limited availability of these measures in HABS. Overall, these results indicate that models based on clinical variables, APOE4 and hippocampal volume maintain good discrimination in an independent cohort, supporting their generalizability.

### Final selection of the predictive models

We finally selected five models for practical use and deployed them as web applications.

The clinical model is suitable for use in general neurology or geriatric settings. Developed in 613 participants with 123 events, it demonstrated acceptable discrimination, excellent calibration, and consistent performance in internal and external validation.

The models incorporating hippocampal volume and plasma NfL levels are better suited to memory clinics, as they require access to non-routine data. Both models obtained higher C-index values than the clinical model, while maintaining good calibration. The hippocampal model was validated as robustly as the clinical model, whereas the NfL model was developed in 272 participants with 59 events and was not externally validated.

The models including CSF tau/Aβ42 and FDG and florbetapir PET are primarily applicable in research settings. These models demonstrated superior discrimination compared with the previous models, but their robustness is limited by smaller training samples and the absence of external validation.

### Kaplan–Meier cumulative incidence curves for cognitive impairment by risk group

**Fig. 2:**
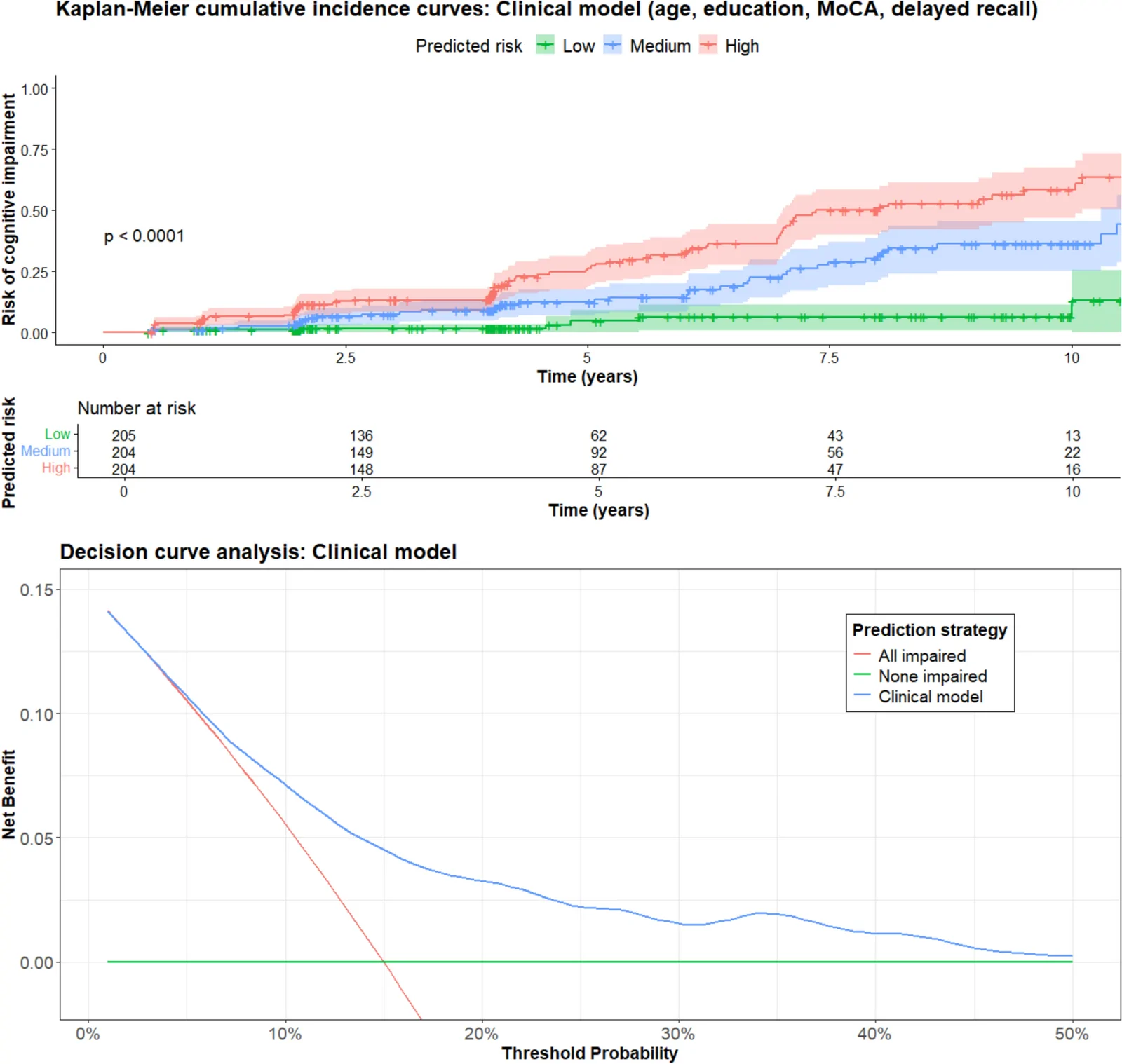
Upper panel: Kaplan–Meier cumulative incidence curves for cognitive impairment stratified by risk groups defined by the clinical model (age, education, MoCA, delayed recall). Lower panel: Decision curve analysis (DCA) of the clinical model

**Fig. 3:**
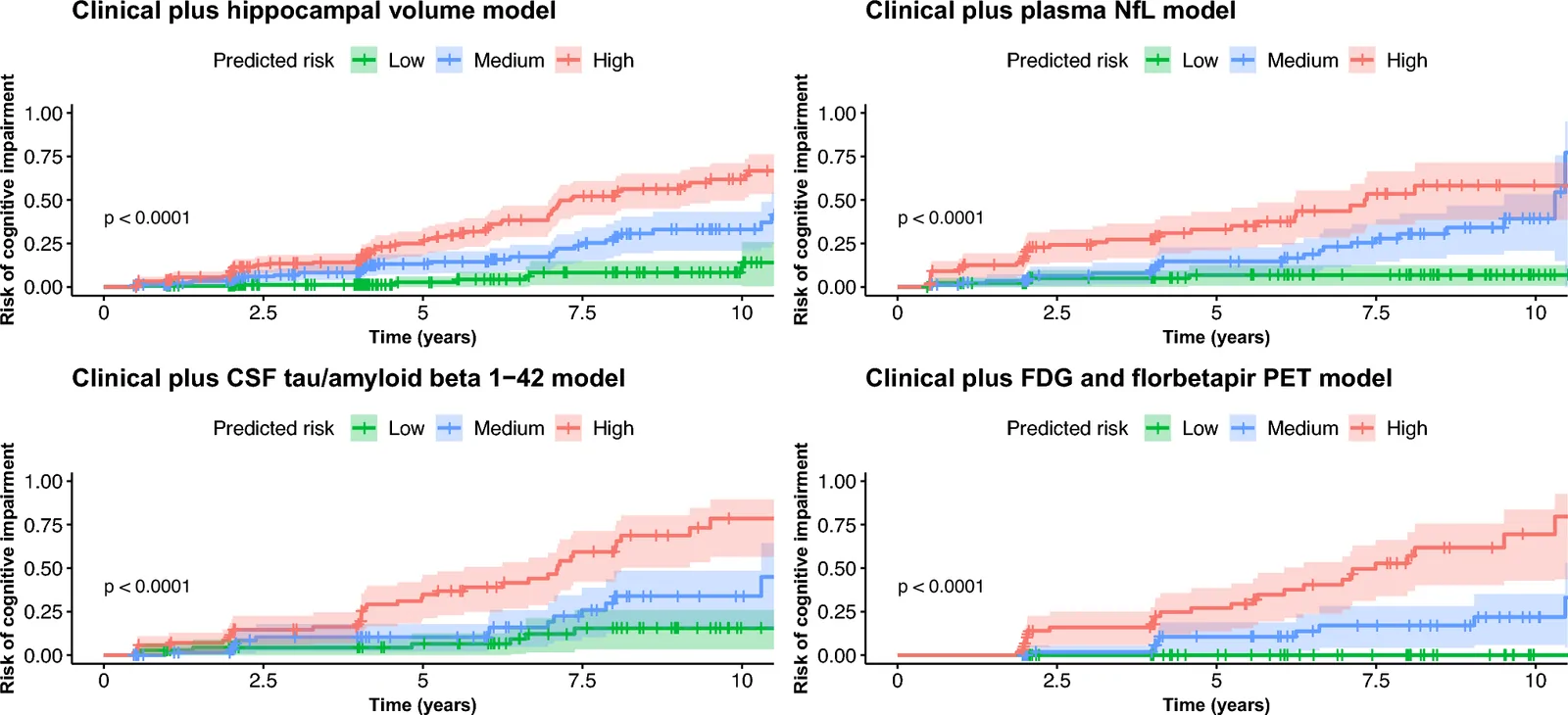
Kaplan–Meier cumulative incidence curves of cognitive impairment, stratified by the risk groups defined by the models including clinical data (age, education, MoCA, delayed recall) plus hippocampal volume, plasma NfL, CSF tau/Aβ42, or FDG and florbetapir PET uptake

Across all five models, stratification by tertiles of the linear predictor produced clear separation of cumulative incidence curves (log-rank p < 0.0001) (Fig. 2, upper panel; Fig. 3). Low risk groups showed a persistently low incidence of cognitive impairment in all cases. Models incorporating biomarkers demonstrated earlier and more pronounced divergence between risk groups than the clinical model.

### Decision curve analysis

The clinical model showed a higher net benefit than the default strategies across most of the tested risk thresholds. At very low risk thresholds its performance overlapped with the “all impaired” strategy (Fig. 2, lower panel).

**Fig. 4:**
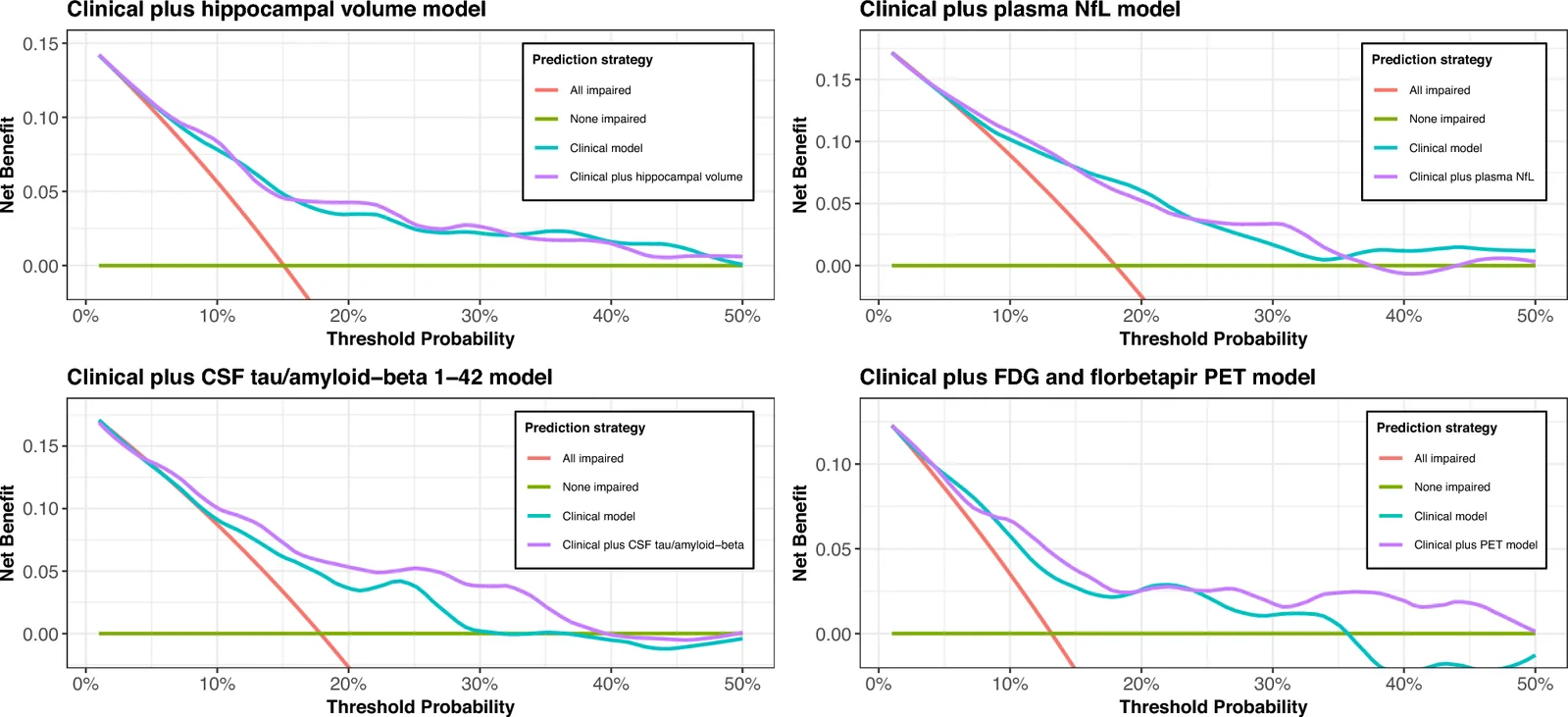
Decision curve analysis of the models including clinical data (age, education, MoCA, delayed recall) combined with hippocampal volume, plasma NfL, CSF tau/Aβ42, or FDG and florbetapir PET uptake. The curves of the clinical-only models fitted to the corresponding datasets are also shown for comparison

The models including hippocampal volume and plasma NfL showed only minor differences compared with the clinical model. In contrast, the models including CSF tau/Aβ42 or FDG and florbetapir PET uptake were clearly superior to the clinical model, particularly at higher risk thresholds (Fig. 4).

### CogStrat web applications

Based on the selected models, we developed five web applications, which can be accessed at the following links:

https://ajimenezhuete.shinyapps.io/CogStrat_Clin/

https://ajimenezhuete.shinyapps.io/CogStrat_Hippo/

https://ajimenezhuete.shinyapps.io/CogStrat_NfL/

https://ajimenezhuete.shinyapps.io/CogStrat_CSF/

https://ajimenezhuete.shinyapps.io/CogStrat_PET/

**Fig. 5:**
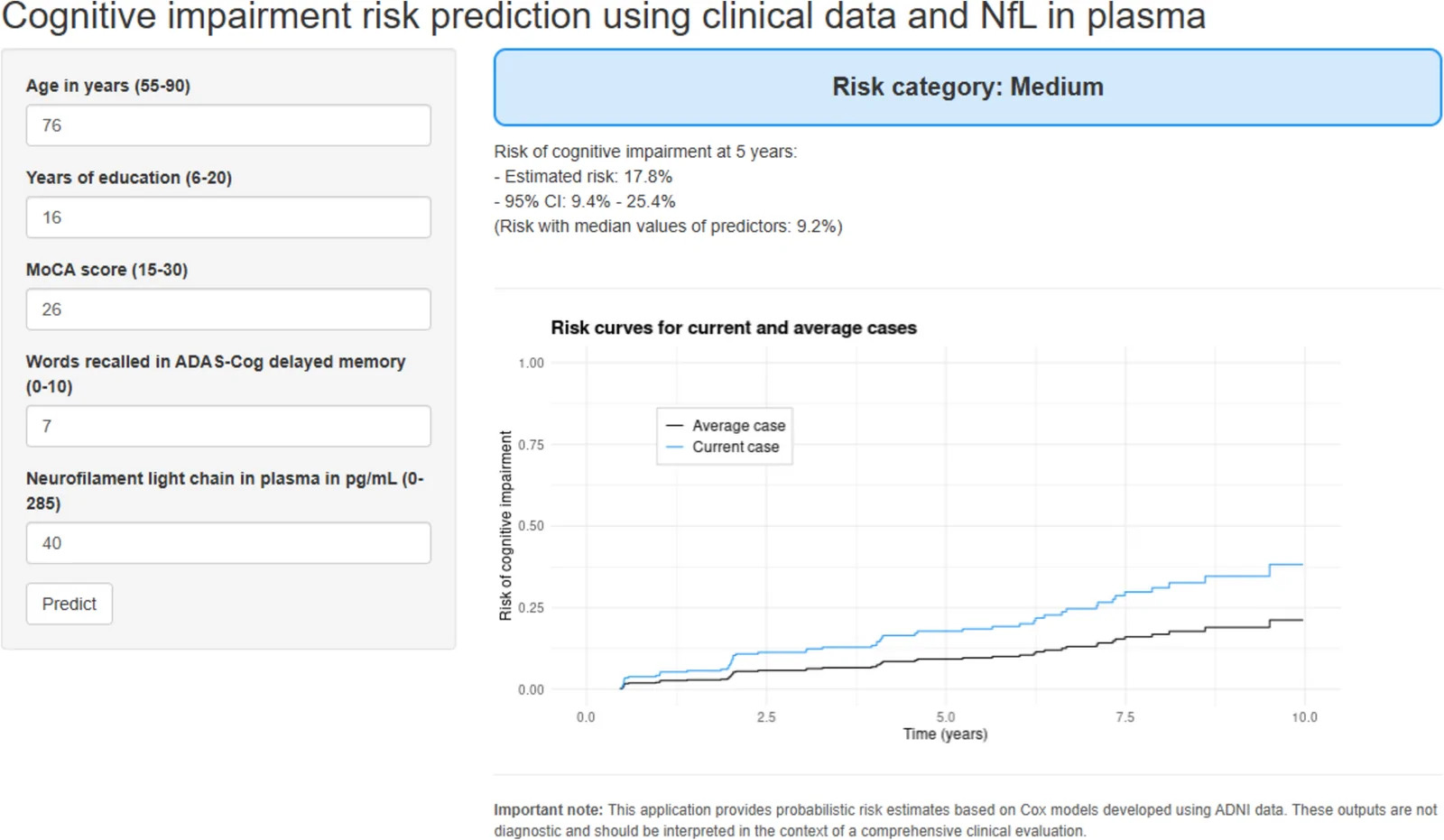
Screenshot of CogStrat_NfL, a web application for risk prediction of incident cognitive impairment in cognitively normal older adults using clinical data and neurofilament light chain (NfL) levels in plasma. The left panel shows predictor inputs, and the right panel shows the risk group classification (low, medium, high), the 5-year risk with 95% confidence interval, and the 10-year cumulative incidence trajectories for the current case and an average case

Users only need an internet connection and a web browser. The initial screen displays input fields for predictor values, with default settings corresponding to their median values. After clicking the Predict button, the results appear on the right side of the screen (Fig. 5).

The R code and associated files required to run the applications locally are available upon request from the corresponding author (AJH).

## Discussion

In this study, we developed and evaluated Cox regression models to predict incident cognitive impairment in cognitively normal older adults. Using longitudinal data from ADNI, with external validation in HABS, we found that a parsimonious clinical model including age, education, MoCA score, and delayed word recall reliably stratifies individuals into low, medium, and high risk groups over a 10-year horizon. The addition of selected biomarkers provided incremental improvements in model discrimination. These models are intended to support clinical practice and should not be interpreted as diagnostic tests.

Using a pre-specified strategy, we selected five models organized in a tiered framework reflecting increasing complexity. At the most accessible level, the clinical model demonstrated acceptable discrimination, excellent calibration, and consistent internal and external validation, supporting its use in general neurology or geriatric settings. At an intermediate level (e.g., memory units), models including hippocampal volume or plasma NfL improved discrimination but required non-routine data. The hippocampal model showed robust validation, whereas the plasma NfL model, developed in a smaller dataset without external validation, warrants more cautious interpretation. At the most complex level, models including CSF tau/Aβ42 or FDG and florbetapir PET achieved the highest discrimination but were limited by smaller samples, reduced accessibility, and lack of external validation, restricting their applicability to research contexts.

To facilitate practical use, the models were deployed as web applications (CogStrat), providing individualized risk group classifications, 5-year risk estimates, and 10-year cumulative incidence curves. From a clinical perspective, CogStrat tools provide actionable information for risk stratification in cognitively normal older adults. In particular, they may support decisions regarding the intensity of longitudinal follow-up, prioritization of individuals for referral to specialized memory clinics, and identification of candidates for preventive interventions or clinical trials. The ability to identify individuals at persistently low risk may also help avoid unnecessary investigations and provide reassurance in routine clinical practice. All authors are clinicians (neurologists and clinical neuropsychologists), and model development was guided by considerations of clinical relevance, feasibility, and interpretability in real-world settings.

Prior models for predicting cognitive impairment in cognitively normal populations vary substantially in study design, predictors, and validation strategies, as highlighted in systematic reviews by Stephan and colleagues (Stephan et al. 2010; Tang et al. 2015; Brain et al. 2024) and in a Cochrane Database review (Mohanannair Geethadevi et al. 2023). Importantly, much of the existing literature has focused on predicting progression from MCI to dementia, where predictive performance is typically higher due to the more advanced disease stage (e.g., Chen et al. 2022; Vermeulen et al. 2025). In contrast, fewer studies have addressed prediction of incident cognitive impairment in cognitively normal individuals, which represents a more challenging but clinically relevant task for early risk stratification.

Earlier models were frequently derived from retrospective or cross-sectional data, whereas more recent studies increasingly rely on prospective longitudinal cohorts. Our models were developed in ADNI and externally validated in HABS, two well-characterized longitudinal studies with standardized cognitive and biomarker assessments.

Sample size and duration of follow-up have also influenced the robustness of published models. Several earlier studies were limited by relatively small sample sizes or short follow-up periods, whereas more recent work has benefited from larger cohorts and longer observation times. Consistent with methodological recommendations, we place the greatest confidence in the clinical and hippocampal volume models, which were developed using sufficiently large samples (> 500), adequate numbers of events (> 100), and conservative predictor-to-event ratios (< 10) (Steyerberg 2019).

Modeling strategies in this field have evolved from predominantly logistic regression approaches toward greater use of time-to-event models and, more recently, machine learning methods. While machine learning approaches can provide incremental improvements in predictive performance, they often reduce interpretability in clinical settings and increase the risk of overfitting (Steyerberg 2019). Our use of Cox PH regression reflects a deliberate trade-off favoring transparency and clinical applicability.

Reporting of model performance has improved over time, yet important gaps remain (Stephan et al. 2010; Tang et al. 2015; Mohanannair Geethadevi et al. 2023; Brain et al. 2024). Although discrimination metrics such as the AUC or C-index are now routinely reported, calibration is still frequently underreported despite its critical importance for clinical decision-making. In this study, we systematically reported both discrimination and calibration and evaluated the incremental value of biomarkers using clinically oriented metrics, including the NRI and DCA, which often tempered the improvements suggested by classical measures of model fit. Reported C-index/AUC values in this field typically range from 0.60 to 0.70, reinforcing the limitations of precise absolute risk prediction and supporting our emphasis on risk group stratification.

Validation practices have similarly improved over time, although external validation remains relatively uncommon, with many models evaluated only internally (Stephan et al. 2010; Tang et al. 2015; Mohanannair Geethadevi et al. 2023; Brain et al. 2024). This limitation raises concerns regarding generalizability across populations and clinical settings. By incorporating both internal and external validation for key models, our study addresses a frequent limitation of earlier work. Nevertheless, external validation was not feasible for all biomarker-based models due to differences in data availability, highlighting the challenges of developing broadly generalizable multimodal prognostic tools.

Beyond individual model validation, systematic external validation and head-to-head comparison of multiple prediction models represents an important direction for future research. The relevance of this need has been commented in other fields, particularly on cardiovascular risk prediction (Damen et al. 2016), but remain relatively limited in dementia research, especially for biomarker-based models. Recent studies comparing established dementia risk scores illustrate both the feasibility and the variability of model performance across populations (Huque and Anstey 2025; Stubs et al. 2025). Conducting such analyses requires access to harmonized longitudinal datasets containing all relevant predictors, which represents a substantial methodological challenge.

With respect to predictor selection, early models relied primarily on demographic and cognitive variables, whereas later studies increasingly incorporated genetic, vascular, lifestyle, and biomarker data (Stephan et al. 2010; Tang et al. 2015; Mohanannair Geethadevi et al. 2023; Brain et al. 2024). Recent studies have reported associations between plasma biomarkers and future cognitive decline in cognitively unimpaired adults (Mattsson-Carlgren et al. 2023). Yakoub et al. (2025) showed that plasma p-tau217 can stratify long-term risk of progression to MCI, with strong group-level discrimination. However, their analyses did not evaluate calibration or the incremental value of p-tau217 beyond cognitive measures. In contrast, although p-tau217 was a significant univariate predictor in our study, its inclusion in multivariable models provided little improvement in discrimination and was associated with poor calibration. Finally, our results highlight the superior prognostic value of the MoCA compared with the MMSE, supporting its use beyond diagnostic screening.

Several prediction models have been implemented as dementia risk calculators, primarily within public health and prevention frameworks. The CAIDE score was derived from a population-based midlife cohort and is widely used in epidemiological research (Kivipelto et al. 2006). The ANU-ADRI and CogDrisk were explicitly designed as self-report calculators for community-dwelling adults, emphasizing modifiable lifestyle and vascular factors (Anstey et al. 2013, 2022). Similarly, the LIBRA index quantifies modifiable risk factors and has been applied mainly in prevention research (Schiepers et al. 2018). In contrast, CogStrat applications are designed to translate prognostic models into tools that support clinical practice, enabling risk stratification and guiding triage for longitudinal monitoring.

Several limitations should be acknowledged. First, both ADNI and HABS are volunteer-based cohorts enriched for AD risk, which may limit generalizability to broader or more diverse populations. Second, the ADNI cohort primarily focuses on amnestic, late-onset AD, which may reduce the applicability of our findings to atypical AD presentations or non-AD dementias. Third, limited sample sizes for certain biomarker-specific datasets restricted validation and may constrain the clinical applicability of some high-performing models. Fourth, missing and non-overlapping biomarker data precluded construction of multimodal models combining several biomarkers. Finally, although the models estimate long-term risk, they remain probabilistic and have not been prospectively tested for clinical decision-making impact. Further validation in population-based, more diverse, and non-research cohort samples is needed to confirm generalizability across different settings.

In conclusion, CogStrat provides a framework for long-term prediction of cognitive impairment in older adults that can be adapted to different contexts. By translating statistical models into freely accessible, interactive tools, it represents a pragmatic step toward integrating longitudinal risk modeling into routine clinical practice, enabling personalized monitoring and targeted follow-up.

## Supplementary Information

Below is the link to the electronic supplementary material.

Supplementary file1 (DOCX 11 KB)

Supplementary file2 (DOCX 3023 KB)

## Data Availability

The ADNI and HABS data can be accessed upon request and approval by the ADNI and HABS evaluation committees.
