# Supplementary material for: CogStrat: web-based tools for predicting incident cognitive impairment in cognitively normal older adults
Source: J Neural Transm (Vienna). 2026 May 26;133(9):2371–85. doi: 10.1007/s00702-026-03163-7 (PMC13687235; doi:10.1007/s00702-026-03163-7)
Supplement: Supplementary file 1 [file 702_2026_3163_MOESM1_ESM.docx]

**Supplementary Table 1**

Main characteristics of the Alzheimer’s Disease Neuroimaging Initiative (ADNI) subsets used to fit the Cox models described in Tables 2 and 3

| **Dataset** | **n** | **Events** | **Follow-up (days)** | **Age (years)** | **Sex (male)** | **Education (years)** | **MMSE** |
| --- | --- | --- | --- | --- | --- | --- | --- |
| Clinical data | 613 | 123 | 1496 (790, 2631) | 72  (68, 76) | 272 (44%) | 16  (15, 18) | 29  (29, 30) |
| *APOE* ε4 carrier | 602 | 122 | 1498 (800, 2667) | 72 (68, 77) | 265 (44%) | 16  (15, 18) | 29  (29, 30) |
| Polygenic hazard score | 351 | 95 | 2199 (1452, 3238) | 74 (70, 78) | 174 (50%) | 16  (15, 18) | 29  (29, 30) |
| p-tau217, p-tau21/Aβ42 or Aβ42/Aβ40 in plasma | 166 | 53 | 1450 (778, 1570) | 71 (67, 75) | 68  (41%) | 16  (15, 18) | 30  29, 30) |
| NfL in plasma | 272 | 59 | 1620  (738, 2928) | 72  (68, 77) | 126 (46%) | 16  (15, 18) | 29  29, 30) |
| p-tau181 or tau in CSF | 303 | 75 | 2176  (781, 2993) | 73  (69, 78) | 143 (47%) | 16  (15, 18) | 29  (29, 30) |
| Aβ42, tau/Aβ42 or p-tau181/Aβ42 in CSF | 212 | 58 | 2044  (750, 2919) | 73  (70, 78) | 99  (47%) | 16  (15, 18) | 29  (29, 30) |
| Hippocampal volume | 554 | 111 | 1488  (791, 2609) | 72  (68, 76) | 246 (44%) | 16  (15, 18) | 29  (29, 30) |
| FDG-PET | 332 | 80 | 1991  (756, 2998) | 73  (69, 78) | 161 (48%) | 16  (15, 18) | 29  (28, 30) |
| Florbetapir-PET | 242 | 53 | 1893  (865, 2956) | 72  (69, 77) | 107 (44%) | 16  (15, 18) | 29  (29, 30) |
| FDG and florbetapir PET | 181 | 38 | 2035  (1444, 2992) | 72  (69, 77) | 85  (47%) | 16  (15, 18) | 29  (29, 30) |

Notes: Quantitative variables are summarized as median (interquartile range). Qualitative variables are summarized as frequency (percentage). The events indicate the number of participants who develop cognitive impairment (mild cognitive impairment or dementia)

Abbreviations: Aβ40, amyloid beta 1-40; Aβ42, amyloid beta 1-42; C-index, concordance index; 95% CI, 95% bootstrap confidence interval; CSF, cerebrospinal fluid; FDG, [¹⁸F]fluorodeoxyglucose; LRT, likelihood ratio test; NRI, net reclassification index; MMSE, Mini-Mental State Examination; n, sample size; PET, positron emission tomography; p-tau181, phosphorylated tau at threonine 181; p-tau217, phosphorylated tau at threonine 217
