# Supplementary material for: CogStrat: web-based tools for predicting incident cognitive impairment in cognitively normal older adults
Source: J Neural Transm (Vienna). 2026 May 26;133(9):2371–85. doi: 10.1007/s00702-026-03163-7 (PMC13687235; doi:10.1007/s00702-026-03163-7)
Supplement: Supplementary file 2 [file 702_2026_3163_MOESM2_ESM.docx]

**Supplementary Table 2**

Characteristics of the Harvard Aging Brain Study (HABS) sample of cognitively unimpaired participants used for external validation (n = 287), stratified by conversion to cognitive impairment (mild cognitive impairment or dementia)

| **Variable** | **Conversion = No** | **Conversion = Yes** | **All participants** |
| --- | --- | --- | --- |
|  | n = 264 | n = 23 | n = 287 |
| Time of follow-up (days) | 1830 (1576, 1903) | 1464 (801, 1845) | 1826 (1535, 1900) |
| Age (years) | 78 (73, 83) | 80 (76, 84) | 78 (73, 83) |
| Sex (male) | 112 (42%) | 6 (26%) | 118 (41%) |
| Education (years) | 16 (13, 18) | 16 (14, 18) | 16 (13, 18) |
| MMSE (0-30) | 29 (28, 30) | 28 (27, 29) | 29 (28, 30) |
| Delayed word recall (0-10) | 6 (4, 7) | 7 (5, 9) | 6 (4, 8) |
| TMT-A (seconds) | 34 (27, 42) | 40 (31, 53) | 35 (27, 43) |
| Digits forward | 7 (6, 8) | 7 (6, 8) | 7 (6, 8) |
| Digits backwards | 5 (4, 6) | 5 (4, 6) | 5 (4, 6) |
| FAS (F letter) | 15 (12, 18) | 16 (183, 20) | 15 (12, 18) |
| *APOE* ε4 carrier | 69 (26%) | 10 (43%) | 79 (28%) |
| Hippocampal volume (µL) | 7465 (6908, 7918) | 6851 (6396, 7232) | 7412 (6841, 7901) |
| - Missing | 8 | 1 | 9 |
| PET with FDG (SUVR) | 1.4 (1.3, 1.6) | 1.4 (1.3, 1.5) | 1.4 (1.3, 1.5) |
| - Missing | 4 | 1 | 5 |
| PET with PiB (SUVR) | 11.2 (7.9, 12.2) | 12.9 (10.7, 16.5) | 11.3 (8.0, 12.3) |
| - Missing | 2 | 1 | 3 |

Notes: Descriptive statistics are given as median (interquartile range) or frequency (percentage). Delayed word recall corresponds to the number of words recalled in the ADAS-Cog task. Hippocampal volume indicates the bilateral hippocampal volume calculated with MRI. PET with FDG is a measurement of FDG uptake at the angular, temporal and posterior cingulate regions. PET with PiB is a measurement of Pittsburgh compound B uptake

Abbreviations: ADAS-Cog, Alzheimer's Disease Assessment Scale-Cognitive subscale; FAS, FAS Phonemic Verbal Fluency test; FDG, [18F]Fluorodeoxyglucose; MMSE, Mini-Mental State Examination; PET, positron emission tomography; PiB, Pittsburgh B compound; SUVR, standardized uptake value ratio; TMT-A, Trail Making Test part A
